# The alkaline phosphatase-to-prealbumin ratio combined with CT-quantified sarcopenia predicts survival in gastric cancer patients after surgical resection

**DOI:** 10.3389/fnut.2025.1636230

**Published:** 2025-09-30

**Authors:** Yunxin Xu, Zhongze Du, Yingwei Xue, Hongjiang Song

**Affiliations:** Department of Gastrointestinal Surgery, Harbin Medical University Cancer Hospital, Harbin Medical University, Harbin, Heilongjiang, China

**Keywords:** sarcopenia, surgery, gastric cancer, prognosis, biomarker

## Abstract

**Objectives:**

The aim of this study was to comparatively evaluate the prognostic efficacy of alkaline phosphatase to prealbumin ratio (APR) against eight established hematological biomarkers. This study pioneered the development of APR-sarcopenia, a novel composite biomarker integrating hematological indices with computed tomography-quantified body composition parameter, and assessed its predictive performance.

**Methods:**

This study included 190 gastric cancer patients who underwent surgery and had computed tomography (CT) scans at our institution between January 2016 and December 2017. Comprehensive clinical data were available for all patients. Differences in clinical and pathological characteristics were analyzed using the Chi-square test, Fisher's exact test, one-way ANOVA, and the Kruskal-Wallis test. Survival differences were evaluated using Kaplan–Meier survival curves and the log-rank test. Cox regression analysis was performed to identify independent prognostic factors, and nomograms were constructed to predict survival probabilities.

**Results:**

Patients were divided into three groups based on sarcopenia and APR levels: Group 1 (80 cases), Group 2 (65 cases), and Group 3 (45 cases). Patients in Group 3 had longer progression-free survival (PFS) (HR = 0.403, *p* < 0.001) and overall survival (OS) (HR = 0.394, *p* < 0.001). APR-sarcopenia had the highest area under the curve (AUC) among all biomarkers, with predictive accuracy approaching that of the TNM staging. Multivariate Cox regression analysis identified pTNM, CA724, and APR-sarcopenia as independent prognostic factors for both PFS and OS. The nomograms for PFS and OS had C-index values of 0.794 (95% CI: 0.743–0.845) and 0.801 (95% CI: 0.751–0.851), respectively. Calibration analysis confirmed that the nomograms accurately predicted 3- and 5-year survival rates for PFS and OS.

**Conclusions:**

APR exhibited superior prognostic accuracy for postoperative outcomes in gastric cancer patients compared to other hematological biomarkers. APR-sarcopenia demonstrated enhanced prognostic value and served as an independent prognostic marker. Additionally, APR-sarcopenia can help identify patients at high risk of metastasis and recurrence following gastric cancer surgery.

## Introduction

Over the past few decades, the incidence of gastric cancer has significantly declined in the United States and Western Europe. Nevertheless, it remains a major global health concern, especially in East Asian countries. In 2020, gastric cancer accounted for over 1 million cases and more than 768,000 deaths worldwide, ranking as the fifth most frequently diagnosed cancer and the third leading cause of cancer-related mortality (1). Despite the emergence of various treatment modalities, including chemotherapy, radiotherapy, and immunotherapy, surgery remains the only curative approach for gastric cancer (2). For patients with early-stage gastric cancer who undergo radical surgery combined with chemotherapy, the 5-year survival rate is 90%. However, due to the absence of specific symptoms in early gastric cancer and its low detection rate, over 70% of patients progress to advanced-stage gastric cancer. Even those who undergo surgery for advanced gastric cancer remain at high risk of metastasis and recurrence (3). Consequently, there is an urgent need for reliable biomarkers to predict postoperative survival outcomes, facilitate early identification of high-risk patients and enable timely therapeutic interventions.

Given the widespread clinical application of blood testing, researchers have identified biomarkers with robust predictive capabilities based on hematological parameters from gastric cancer patients. The biomarkers of the systemic inflammatory response, such as systemic immune-inflammation index (SII) (4, 5) and systemic inflammation response index (SIRI) (6), have been demonstrated to possess strong prognostic value in gastric cancer. Prognostic nutritional index (PNI), a novel biomarker of nutritional status, has also been validated as a potential predictor of survival, recurrence, and prognosis of gastric cancer patients (7, 8). First proposed in 2020, alkaline phosphatase to prealbumin ratio (APR) has emerged as an independent prognostic biomarker, exhibiting superior predictive performance compared to established biomarkers including the lymphocyte-to-monocyte ratio (LMR), platelet-to-lymphocyte ratio (PLR), and neutrophil-to-lymphocyte ratio (NLR) (9).

Sarcopenia, a complex syndrome characterized by the progressive and generalized loss of skeletal muscle mass and function, was redefined in 2018 by the European Working Group on Sarcopenia in Older People (EWGSOP). The updated diagnostic criteria emphasize reduced muscle quantity or quality as essential diagnostic components. Computed tomography (CT) and magnetic resonance imaging (MRI) remain the gold standard for muscle mass quantification (10). In patients with cancer cachexia, anorexia, malnutrition, and systemic inflammation, an intensified metabolic state contributes to the development of sarcopenia (11). Recent studies have demonstrated an association between sarcopenia and various cancers, including gastric cancer (12), liver cancer (13), and lung cancer (14).

In summary, developing a predictive biomarker incorporating CT imaging and hematological parameters obtained upon hospital admission holds significant clinical value for predicting postoperative prognosis in gastric cancer patients. This study aims to comparatively assess the prognostic performance of APR against eight established biomarkers in gastric cancer patients, while proposing a novel composite indicator that combines post-admission CT-quantified body composition parameters and hematological profiles to enhance prognostic accuracy.

## Materials and methods

### Patients

We enrolled 190 gastric cancer patients who underwent surgery at our institution between January 2016 and December 2017. The inclusion criteria were: (1) Patients diagnosed with gastric cancer who had undergone surgical treatment; (2) Patients who had undergone abdominal CT scans at our institution. The exclusion criteria were: (1) Patients with severe cardiovascular disease, chronic kidney disease, chronic liver disease, chronic pulmonary disease, or autoimmune disorders; (2) Patients in an acute inflammatory state; (3) Patients with gastric cancer combined with other primary malignant tumors; (4) Patients with liver or bone disorders known to influence ALP levels. (5) Patients with incomplete clinical data.

### Data collection

Patients were followed up by telephone or outpatient visits every 3–6 months for the first 2 years, every 6–12 months for the third to fifth years, and annually thereafter. The study's primary endpoints were progression-free survival (PFS) and overall survival (OS). PFS was defined as the period from the first day of surgery to the date of disease progression, death, withdrawal from the follow-up, or the time of the last follow-up. Disease progression was confirmed through comprehensive imaging or pathological examinations. Overall survival was defined as the duration from the initiation of treatment to the date of death, withdrawal from follow up, or the last follow-up. Clinical and pathological data of patients were collected through the hospital's electronic medical record system.

### Sarcopenia and APR

**Figure 1:**
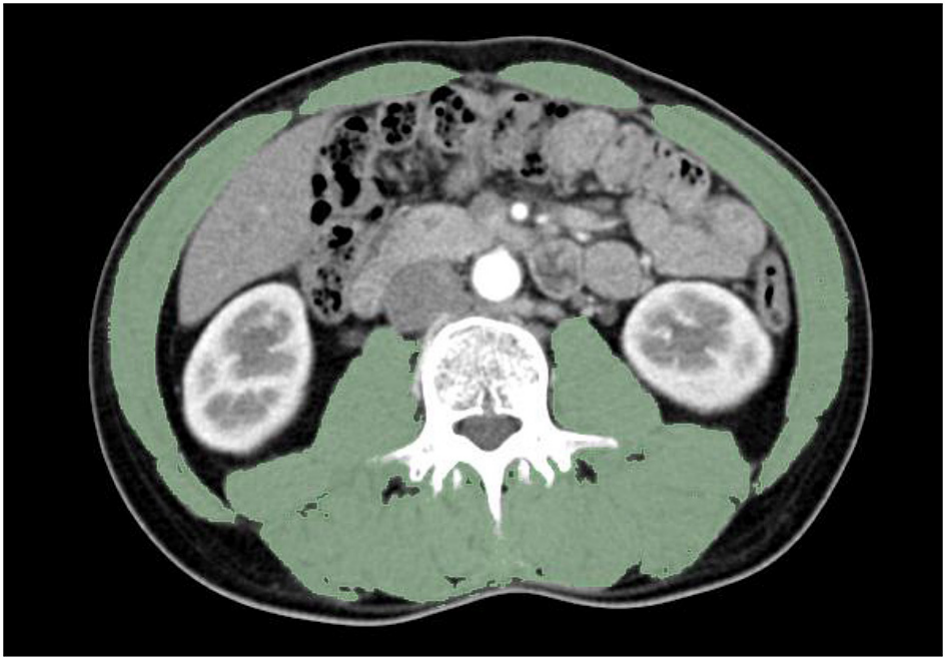
Example of a computed tomography image.

A radiologist with more than ten years of experience, who was blinded to the clinical outcomes of the participants, interpreted the CT scans using imaging software to assess skeletal muscle area (SMA), subcutaneous fat area (SFA) and visceral fat area (VFA) (Figure 1). The CT data for each patient were uploaded into 3D Slicer (Version 4.10.2) for a detailed analysis of the cross-sectional area of skeletal muscles at the third lumbar vertebra (L3), measured in Hounsfield units (HU) across the entire muscle region. The HU threshold range for skeletal muscle was defined as ranging from −29 to 150. The skeletal muscle index (SMI) at L3 was calculated by dividing the skeletal muscle area by the square of the patient's height. Currently, there is a lack of definitive diagnostic criteria for sarcopenia, so we used receiver operating characteristic (ROC) analysis to determine the optimal cut-off values for SMI and APR. In this cohort, women with SMI less than 31.40 cm^2^/m^2^ and men with SMI less than 39.26 cm^2^/m^2^ were diagnosed with sarcopenia. Additionally, the optimal cut-off value for APR was 0.26. Patients with sarcopenia and APR ≥ 0.26 were assigned to Group 1, those without sarcopenia and with APR < 0.26 were assigned to Group 3, and the remaining patients were assigned to Group 2.

### Statistical analysis

All statistical analyses were performed using IBM SPSS Statistics 25 (Chicago, IL, USA), R 4.3.3 (Vienna, Austria) and GraphPad Prism 10 (La Jolla, CA, USA), with statistical significance defined as a two-sided *p* < 0.05. Continuous variables with normal distribution were presented as mean ± standard deviation (SD), while non-normally distributed variables were expressed as median with interquartile range (IQR). Categorical data were summarized as frequencies and percentages. Comparative analyses of clinicopathological characteristics were conducted using appropriate parametric and non-parametric tests: one-way ANOVA or Kruskal-Wallis test for continuous variables, and Chi-square test or Fisher's exact test for categorical variables, depending on data distribution and cell frequency requirements. Survival curves were generated using the Kaplan-Meier method, and differences between the curves were evaluated with the log-rank test. Cox regression analysis was employed to identify independent prognostic factors and select the variables to be included in the nomograms. Subsequently, nomograms were used to predict the survival probabilities of gastric cancer patients, with accuracy assessed using C-index and calibration curves.

## Results

### Patient characteristics

We enrolled 190 patients, comprising 126 males and 64 females, with a median age of 60 years. There were 124 cases (65.3%) of TNM stages I and II, and 66 cases (34.7%) of TNM stages III and IV. Chi-square test and Fisher's exact test demonstrated significant associations between APR-sarcopenia and both tumor size and TNM stage. Additionally, one-way ANOVA and the Kruskal-Wallis test revealed significant correlations between APR-sarcopenia and various clinical and hematological parameters, including age, body mass index (BMI), albumin (ALB), hemoglobin (Hb), subcutaneous fat area (SFA), and visceral fat area (VFA) (Table 1).

**Table 1:** Patient characteristics.

***n***	**Level**	**Group 1**	**Group 2**	**Group 3**	***p***
		**80**	**65**	**45**	
Sex	Male	50 (62.5)	42 (64.6)	34 (75.6)	0.313
	Female	30 (37.5)	23 (35.4)	11 (24.4)	
Age	Median (IQR)	65.00 (58.00–71.00)	60.00 (53.00–65.00)	52.50 (45.00–60.25)	< 0.001
BMI (kg/m^2^)	Mean ± SD	21.32 ± 3.11	22.37 ± 3.09	24.15 ± 3.14	< 0.001
Borrmann type	I + II	26 (32.5)	22 (33.8)	22 (48.9)	0.157
	III + IV	54 (67.5)	43 (66.2)	23 (51.1)	
Tumor size	< 50 mm	32 (40.0)	37 (56.9)	29 (64.4)	0.018
	≥50 mm + unknown	48 (60.0)	28 (43.1)	16 (35.6)	
pTNM	I + II	44 (55.0)	47 (72.3)	33 (73.3)	0.040
	III + IV	36 (45.0)	18 (27.7)	12 (26.7)	
ALT (U/L)	Median (IQR)	16.25 (13.00–22.00)	19.00 (13.00–27.00)	17.00 (13.00–25.00)	0.746
TBIL (μmol/L)	Median (IQR)	10.15 (8.17–15.39)	10.68 (8.58–13.68)	13.04 (8.67–16.84)	0.183
DBIL (μmol/L)	Median (IQR)	4.11 (2.86–5.30)	3.84 (2.99–4.59)	4.61 (3.33–5.80)	0.092
IDBIL (μmol/L)	Median (IQR)	6.93 (4.87–10.47)	7.20 (5.08–9.71)	9.00 (5.63–11.42)	0.184
TP (g/L)	Median (IQR)	67.00 (61.93–71.00)	68.00 (65.00–72.55)	68.00 (65.10–73.50)	0.174
ALB (g/L)	Median (IQR)	40.00 (37.00–41.80)	41.00 (38.00–44.00)	42.00 (40.00–44.00)	0.001
GLOB (g/L)	Median (IQR)	27.00 (24.85–30.85)	27.00 (24.00–30.00)	26.20 (24.00–30.00)	0.743
PA (mg/L)	Median (IQR)	229.50 (187.00–265.75)	281.00 (233.00–315.50)	308.00 (289.50–371.50)	< 0.001
ALP (U/L)	Median (IQR)	77.00 (68.00–90.75)	71.00 (62.50–89.00)	61.00 (51.00–72.50)	< 0.001
WBC (10^9^/L)	Median (IQR)	6.31 (5.25–7.58)	6.26 (5.20–7.15)	6.63 (5.30–7.73)	0.735
NEU (10^9^/L)	Median (IQR)	3.82 (2.90–4.86)	3.52 (2.75–4.29)	3.47 (2.90–4.27)	0.302
L (10^9^/L)	Median (IQR)	1.72 (1.37–2.15)	2.04 (1.59–2.44)	2.01 (1.54–2.58)	0.058
Mono (10^9^/L)	Median (IQR)	0.47 (0.36–0.61)	0.42 (0.34–0.50)	0.46 (0.35–0.59)	0.175
Hb (10^9^/L)	Median (IQR)	127.95 (111.00–142.30)	138.00 (127.65–151.50)	136.00 (127.30–151.50)	0.001
IgA (g/L)	Median (IQR)	2.32 (1.57–2.98)	2.12 (1.62–2.91)	2.15 (1.42–2.66)	0.463
IgG (g/L)	Median (IQR)	10.65 (8.81–12.55)	10.50 (8.70–12.25)	11.00 (8.72–12.50)	0.971
IgM (g/L)	Median (IQR)	0.81 (0.57–1.09)	0.92 (0.69–1.15)	0.79 (0.57–1.22)	0.361
CEA (ng/mL)	Median (IQR)	2.09 (1.28–4.79)	1.92 (1.02–3.00)	1.87 (0.97–2.99)	0.293
CA199 (U/mL)	Median (IQR)	9.76 (5.52–25.03)	8.87 (4.38–16.74)	9.43 (4.75–12.68)	0.419
CA724 (U/mL)	Median (IQR)	2.05 (1.17–6.55)	2.79 (1.14–6.69)	1.67 (0.90–3.63)	0.208
CA125II (U/mL)	Median (IQR)	10.79 (7.13–16.23)	9.97 (7.80–13.89)	8.33 (6.36–11.92)	0.087
SFA (cm^2^)	Median (IQR)	65.50 (43.20–105.49)	84.78 (62.16–138.73)	93.34 (58.22–124.37)	0.011
VFA (cm^2^)	Median (IQR)	57.97 (20.84–80.51)	64.68 (35.71–98.64)	82.18 (26.55–121.40)	0.020

BMI, body mass index; ALT, alanine aminotransferase; TBIL, total bilirubin; DBIL, direct bilirubin; IDBIL, indirect bilirubin; TP, total protein; ALB, albumin; GLOB, globulin; PA, prealbumin; WBC, white blood cell; NEU, neutrophil; L, lymphocyte; mono, monocyte; Hb, hemoglobin; IgA, immunoglobulin A; IgG, immunoglobulin G; IgM, immunoglobulin M; CEA, carcinoembryonic antigen; CA199, carbohydrate antigen 199, CA724, carbohydrate antigen 724; CA125II, carbohydrate antigen 125II; SAT, subcutaneous fat area; VAT, visceral fat area.

### Prognostic value of APR and other biomarkers

**Figure 2:**
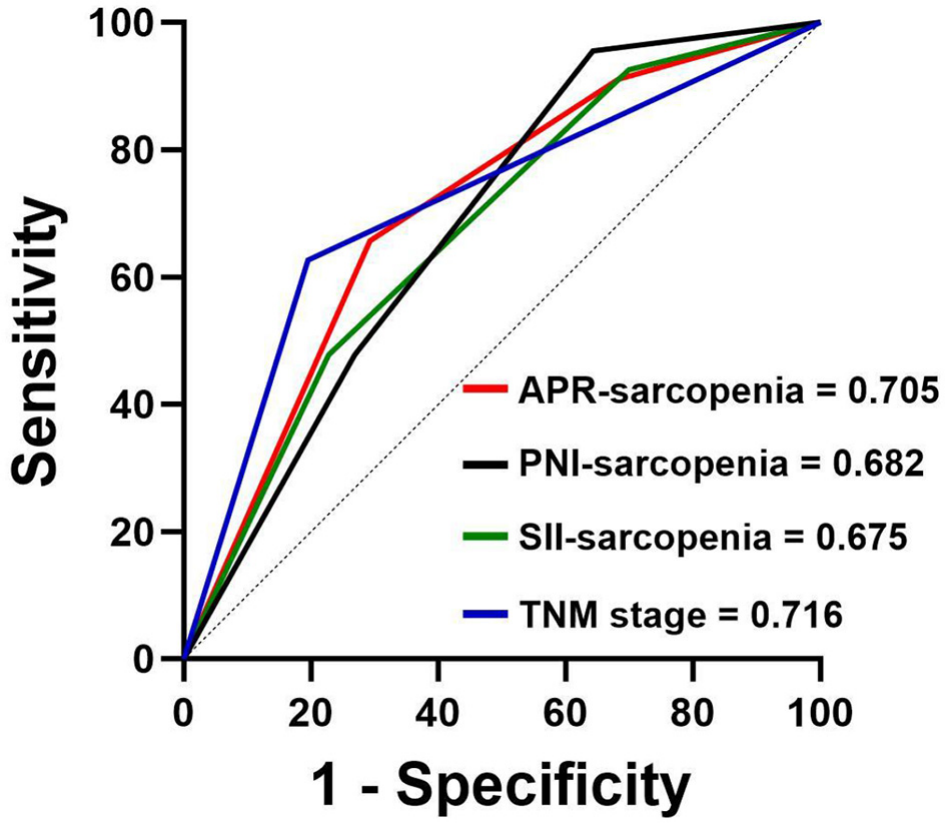
The ROC curves of PNI-sarcopenia, SII-sarcopenia, APR-sarcopenia and TNM stage.

We assessed the prognostic predictive value of APR and eight other nutritional and inflammatory biomarkers, with death as the endpoint. The calculation formulas and results are presented in Table 2. Compared with other hematological composite biomarkers, APR demonstrated superior predictive performance, achieving the highest area under the curve (AUC) of 0.624 (95% CI: 0.543–0.705). Notably, after combining SII and PNI with sarcopenia separately, APR-sarcopenia still exhibited better prognostic capacity, achieving the highest discriminative performance (AUC = 0.705, 95% CI: 0.628–0.781), closely paralleling the TNM stage (AUC = 0.716, 95% CI: 0.636–0.796) (Figure 2).

**Table 2:** The calculation formulas and AUC of hematological composite biomarkers.

**Item**	**Calculation formula**	**AUC**	**95% CI**
GNRI	[1.519 × albumin (g/L)] + [41.7 × (weight/Wlo)]	0.561	0.476–0.646
PNI	albumin (g/dL) + 5 × lymphocyte (10^9^/L)	0.594	0.509–0.678
SII	platelet (10^9^/L) × neutrophil (10^9^/L)/lymphocyte (10^9^/L)	0.571	0.487–0.655
SIRI	monocyte (10^9^/L) × neutrophil (10^9^/L)/lymphocyte (10^9^/L)	0.566	0.481–0.650
ALI	BMI (Kg/m^2^) × albumin (g/dL) × lymphocyte (10^9^/L)/neutrophil (10^9^/L)	0.603	0.520–0.686
NLR	neutrophil (10^9^/L)/lymphocyte (10^9^/L)	0.586	0.502–0.670
PLR	platelet (10^9^/L)/lymphocyte (10^9^/L)	0.614	0.531–0.697
LMR	lymphocyte (10^9^/L)/monocyte (10^9^/L)	0.597	0.513–0.681
APR	alkaline phosphatase (U/L)/prealbumin (mg/L)	0.624	0.543–0.705

The Lorentz equations (Wlo) was as follows: male = Height – 100 – [(Height - 150)/4]; female = Height – 100 – [(Height – 150)/2.5]. GNRI, Geriatric Nutritional Risk Index; PNI, prognostic nutritional index; SII, systemic immune-inflammation index; SIRI, systemic inflammation response index; ALI, advanced lung cancer inflammation index; NLR, neutrophil-to-lymphocyte ratio; PLR, platelet-to-lymphocyte ratio; LMR, lymphocyte-to-monocyte ratio.

### Univariate and multivariate Cox hazard analysis for PFS and OS

To explore the relationship between APR-sarcopenia and prognosis more accurately, we used univariate and multivariate Cox proportional hazards models to identify independent prognostic factors. Univariate analysis identified age (HR = 2.213, 95%CI: 1.311–3.736, *p* = 0.003), Borrmann type (HR = 2.365, 95%CI: 1.347–4.152, *p* = 0.003), tumor size (HR = 2.737, 95%CI: 1.651–4.538, *p* < 0.001), TNM stage (HR = 5.582, 95%CI: 3.379–9.221, *p* < 0.001), lymphocyte count (HR = 0.524, 95%CI: 0.319–0.860, p = 0.011), IgM (HR = 0.595, 95%CI: 0.365–0.971, *p* = 0.038), CA199 (HR = 1.650, 95%CI: 1.012–2.688, *p* = 0.045), CA724 (HR = 2.033, 95%CI: 1.238–3.337, *p* = 0.005) and APR-sarcopenia (*p* < 0.001) as potential prognostic factors for PFS. Similarly, univariate analysis identified age (HR = 2.253, 95%CI: 1.335–3.802, *p* = 0.002), Borrmann type (HR = 2.361, 95%CI: 1.345–4.145, *p* = 0.003), tumor size (HR = 2.704, 95%CI: 1.631–4.483, *p* < 0.001), TNM stage (HR = 5.180, 95%CI: 3.142–8.543, *p* < 0.001), lymphocyte count (HR = 0.519, 95%CI: 0.316–0.851, *p* = 0.009), IgM (HR = 0.587, 95%CI: 0.360–0.957, *p* = 0.033), CA199 (HR = 1.661, 95%CI: 1.018–2.708, *p* = 0.042), CA724 (HR = 2.039, 95%CI: 1.242–3.347, *p* = 0.005) and APR-sarcopenia (*p* < 0.001) as potential prognostic factors for OS. Based on the multivariate regression analysis of variables that were significant in the univariate analysis, TNM stage (HR = 3.881, 95%CI: 2.222–6.779, *p* < 0.001), CA724 (HR = 2.057, 95%CI: 1.187–3.565, *p* = 0.010), and APR-sarcopenia (*p* = 0.001) were confirmed as independent prognostic factors for PFS. In terms of OS, TNM stage (HR = 3.569, 95%CI: 2.072–6.147, *p* < 0.001), CA724 (HR = 2.076, 95%CI: 1.197–3.599, *p* = 0.009), and APR-sarcopenia (*p* < 0.001) were identified as independent prognostic factors (Tables 3, 4).

**Table 3:** Univariate and multivariate analysis for progression-free survival.

**Parameter**	**Univariate analysis**		**Multivariate analysis**	
	**Hazard ratio (95%CI)**	***P*** **value**	**Hazard ratio (95%CI)**	***P*** **value**
Sex (male vs. female)	1.016 (0.610–1.692)	0.952		
Age (< 60 vs. ≥60)	2.213 (1.311–3.736)	0.003	1.334 (0.766–2.321)	0.308
BMI (< 22.07 vs. ≥22.07 kg/m^2^)	0.689 (0.424–1.118)	0.131		
Borrmann type (I + II vs. III + IV)	2.365 (1.347–4.152)	0.003	1.227 (0.642–2.345)	0.537
Tumor size (< 50 vs. ≥50 mm + unknown)	2.737 (1.651–4.538)	< 0.001	1.210 (0.672–2.177)	0.525
pTNM (I + II vs. III + IV)	5.582 (3.379–9.221)	< 0.001	3.881 (2.222–6.779)	< 0.001
ALT (< 17 vs. ≥17 U/L)	0.769 (0.476–1.244)	0.285		
TBIL (< 11.02 vs. ≥11.02 μmol/L)	0.730 (0.451–1.182)	0.201		
DBIL (< 4.09 vs. ≥4.09 μmol/L)	0.994 (0.615–1.604)	0.979		
IDBIL (< 7.30 vs. ≥7.30 μmol/L)	0.655 (0.404–1.063)	0.087		
TP (< 68 vs. ≥68 g/L)	0.962 (0.596–1.554)	0.875		
ALB (< 41 vs. ≥41 g/L)	0.723 (0.447–1.171)	0.188		
GLOB (< 27 vs. ≥27 g/L)	0.861 (0.533–1.391)	0.542		
PA (< 264.5 vs. ≥264.5 mg/L)	0.652 (0.401–1.061)	0.085		
ALP (< 72.5 vs. ≥72.5 U/L)	1.180 (0.730–1.908)	0.499		
WBC (< 6.39 vs. ≥6.39 10^9^/L)	0.748 (0.461–1.212)	0.238		
NEU (< 3.60 vs. ≥3.60 10^9^/L)	1.164 (0.719–1.884)	0.537		
L (< 11.02 vs. ≥11.02 10^9^/L)	0.524 (0.319–0.860)	0.011	0.643 (0.385–1.074)	0.092
Mono (< 0.44 vs. ≥0.44 109/L)	1.071 (0.660–1.737)	0.780		
Hb (< 134.2 vs. ≥134.2 109/L)	0.865 (0.535–1.399)	0.554		
IgA (< 2.22 vs. ≥2.22 g/L)	0.849 (0.525–1.373)	0.505		
IgG (< 10.7 vs. ≥10.7 g/L)	0.825 (0.510–1.334)	0.432		
IgM (< 0.86 vs. ≥0.86 g/L)	0.595 (0.365–0.971)	0.038	0.881 (0.506–1.532)	0.654
CEA (< 1.98 vs. ≥1.98 ng/mL)	1.336 (0.825–2.162)	0.239		
CA199 (< 9.43 vs. ≥9.43 U/mL)	1.650 (1.012–2.688)	0.045	1.462 (0.869–2.458)	0.152
CA724 (< 2.10 vs. ≥2.10 U/mL)	2.033 (1.238–3.337)	0.005	2.057 (1.187–3.565)	0.010
CA125II (< 9.80 vs. ≥9.80 U/mL)	1.409 (0.870–2.281)	0.163		
APR (< 0.26 vs. ≥0.26)	2.261 (1.316–3.884)	0.003		
SFA (< 79.84 vs. ≥79.84 cm^2^)	0.915 (0.567–1.478)	0.717		
VFA (< 63.80 vs. ≥63.80 cm^2^)	0.647 (0.398–1.053)	0.080		
**ARP-sarcopenia**				
Group 1	Ref		Ref	
Group 2	0.392 (0.223–0.686)	0.001	0.440 (0.236–0.820)	0.010
Group 3	0.167 (0.071–0.394)	< 0.001	0.206 (0.084–0.508)	0.001

Results are expressed as hazards ratio (HR) with 95% confidence interval (CI).

**Table 4:** Univariate and multivariate analysis for overall survival.

**Parameter**	**Univariate analysis**		**Multivariate analysis**	
	**Hazard ratio (95%CI)**	**P value**	**Hazard ratio (95%CI)**	**P value**
Sex (male vs. female)	0.980 (0.588–1.632)	0.937		
Age (< 60 vs. ≥60)	2.253 (1.335–3.802)	0.002	1.374 (0.789–2.393)	0.262
BMI (< 22.07 vs. ≥22.07 kg/m^2^)	0.691 (0.425–1.121)	0.134		
Borrmann type (I + II vs. III + IV)	2.361 (1.345–4.145)	0.003	1.289 (0.684–2.432)	0.432
Tumor size (< 50 vs. ≥50 mm + unknown)	2.704 (1.631–4.482)	< 0.001	1.232 (0.689–2.203)	0.481
pTNM (0/Tis + I + II vs. III + IV)	5.180 (3.142–8.543)	< 0.001	3.569 (2.072–6.147)	< 0.001
ALT (< 17 vs. ≥17 U/L)	0.777 (0.481–1.256)	0.303		
TBIL (< 11.02 vs. ≥11.02 μmol/L)	0.720 (0.445–1.167)	0.183		
DBIL (< 4.09 vs. ≥4.09 μmol/L)	1.017 (0.629–1.644)	0.945		
IDBIL (< 7.30 vs. ≥7.30 μmol/L)	0.649 (0.400–1.053)	0.080		
TP (< 68 vs. ≥68 g/L)	0.945 (0.585–1.526)	0.817		
ALB (< 41 vs. ≥41 g/L)	0.702 (0.434–1.138)	0.702		
GLOB (< 27 vs. ≥27 g/L)	0.888 (0.550–1.436)	0.629		
PA (< 264.5 vs. ≥264.5 mg/L)	0.653 (0.402–1.062)	0.086		
ALP (< 72.5 vs. ≥72.5 U/L)	1.236 (0.765–1.999)	0.387		
WBC (< 6.39 vs. ≥6.39 10^9^/L)	0.761 (0.469–1.233)	0.267		
NEU (< 3.60 vs. ≥3.60 10^9^/L)	1.205 (0.744–1.950)	0.448		
L (< 11.02 vs. ≥11.02 10^9^/L)	0.519 (0.316–0.851)	0.009	0.625 (0.371–1.050)	0.076
Mono (< 0.44 vs. ≥0.44 109/L)	1.110 (0.685–1.801)	0.671		
Hb (< 134.2 vs. ≥134.2 109/L)	0.827 (0.511–1.337)	0.438		
IgA (< 2.22 vs. ≥2.22 g/L)	0.840 (0.520–1.359)	0.478		
IgG (< 10.7 vs. ≥10.7 g/L)	0.831 (0.514–1.343)	0.450		
IgM (< 0.86 vs. ≥0.86 g/L)	0.587 (0.360–0.957)	0.033	0.802 (0.461–1.395)	0.435
CEA (< 1.98 vs. ≥1.98 ng/mL)	1.403 (0.866–2.273)	0.169		
CA199 (< 9.43 vs. ≥9.43 U/mL)	1.661 (1.018–2.708)	0.042	1.586 (0.940–2.677)	0.084
CA724 (< 2.10 vs. ≥2.10 U/mL)	2.039 (1.242–3.347)	0.005	2.076 (1.197–3.599)	0.009
CA125II (< 9.80 vs. ≥9.80 U/mL)	1.378 (0.851–2.232)	0.192		
APR (< 0.26 vs. ≥0.26)	2.377 (1.382–4.089)	0.002		
SFA (< 79.84 vs. ≥79.84 cm2)	0.912 (0.565–1.474)	0.707		
VFA (< 63.80 vs. ≥63.80 cm2)	0.652 (0.401–1.059)	0.084		
**APR–sarcopenia**				
Group 1	Ref		Ref	
Group 2	0.381 (0.217–0.668)	0.001	0.435 (0.232–0.813)	0.009
Group 3	0.161 (0.068–0.379)	< 0.001	0.194 (0.079–0.477)	< 0.001

Results are expressed as hazards ratio (HR) with 95% confidence interval (CI).

### Survival analysis for ALI, PLR and LMR

Since advanced lung cancer inflammation index (ALI), platelet-to-lymphocyte ratio (PLR), and lymphocyte-to-monocyte ratio (LMR) demonstrated good discriminative ability in the ROC analysis, we performed survival analyses on these three biomarkers. The optimal thresholds for ALI, PLR, and LMR were determined using ROC analysis, with cut-off values of 413.36, 120.37, and 3.83, respectively.

**Figure 3:**
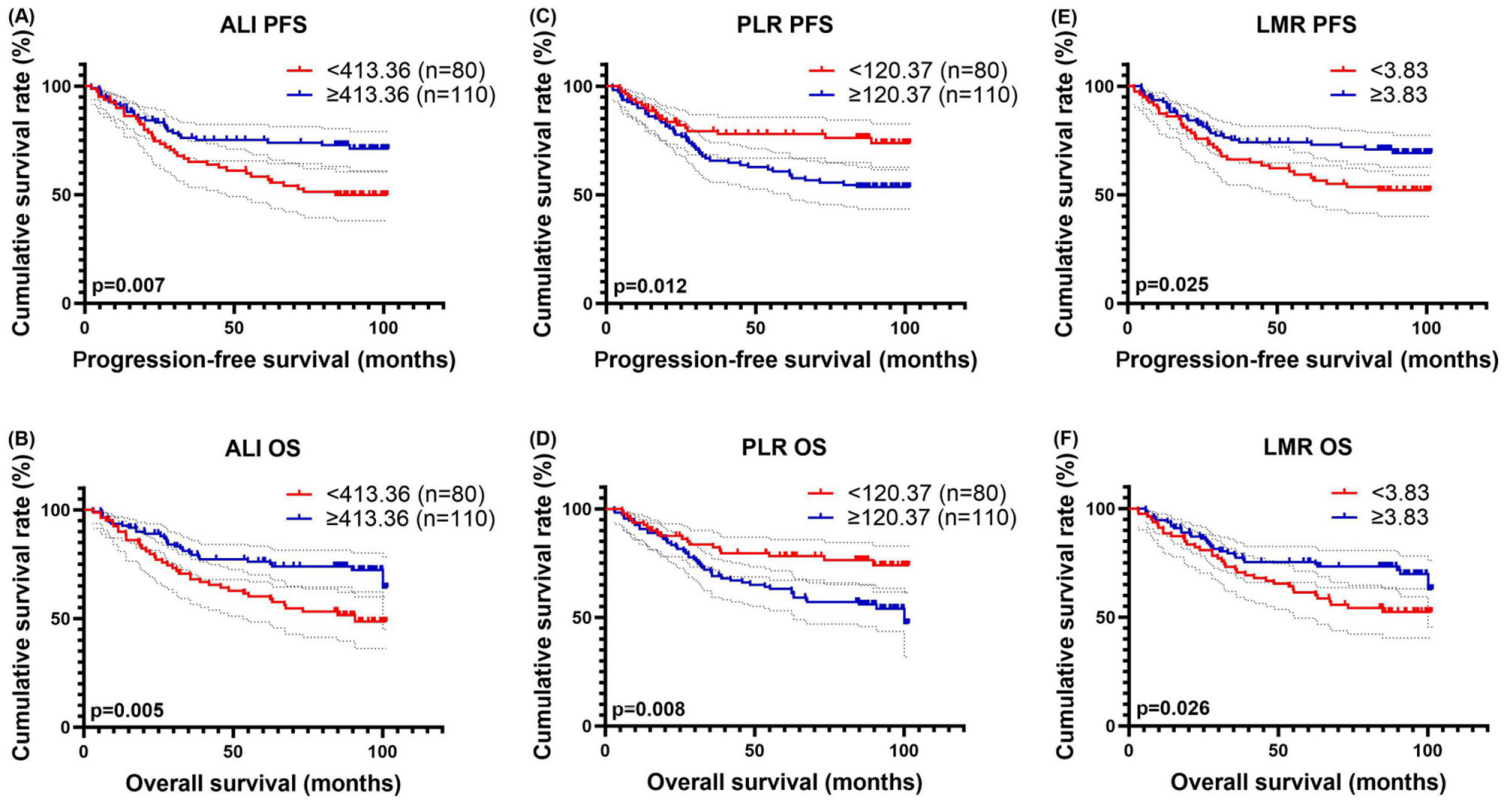
ALI–related survival curve for **(A)** PFS and **(B)** OS, PLR–related survival curve for **(C)** PFS and **(D)** OS, and LMR–related survival curve for **(E)** PFS and **(F)** OS.

Among the 80 patients with ALI < 413.36, the 1-, 3-, and 5-year survival rates were 89.9%, 65.2%, and 58.3% for PFS, and 88.9%, 68.1%, and 60.2% for OS. In contrast, among the 110 patients with ALI ≥ 413.36, the corresponding survival rates were 90.8%, 76.1%, and 75.1% for PFS, and 93.6%, 80.2%, and 76.2% for OS. Patients with higher ALI levels exhibited prolonged PFS (HR = 0.522, *p* = 0.008) and OS (HR = 0.504*, p* = 0.006) (Figures 3A, B).

After grouping, 80 patients were categorized into the PLR < 120.37 group, while 110 patients were assigned to the PLR ≥ 120.37 group. The 1-, 3-, and 5-year survival rates for PFS were 91.3%, 79.3%, and 77.9% in the PLR < 120.37 group, compared to 89.9%, 65.8%, and 60.7% in the PLR ≥ 120.37 group. Likewise, the corresponding OS rates were 93.8%, 83.5%, and 78.1% in the lower PLR group, whereas those in the higher PLR group were 90.8%, 69.0%, and 63.1%. Notably, elevated PLR levels were associated with worse PFS (HR = 1.954, *p* = 0.014) and OS (HR = 2.011, *p* = 0.010) (Figures 3C, D).

There were 79 patients with LMR < 3.83, and their 1-, 3-, and 5-year survival rates for PFS and OS were 87.3%, 66.3%, and 59.3% and 88.6%, 71.9%, and 61.4%, respectively. While there were 111 patients with LMR ≥ 3.83, and their 1-, 3-, and 5-year survival rates for PFS and OS were 92.7%,75.3%, and 74.2% and 94.6%, 77.4%, and 75.4%, respectively. Patients with high LMR levels had longer PFS (HR = 0.581, *p* = 0.027) and OS (HR = 0.582, *p* = 0.028) (Figures 3E, F).

### Survival analysis for APR and sarcopenia

**Figure 4:**
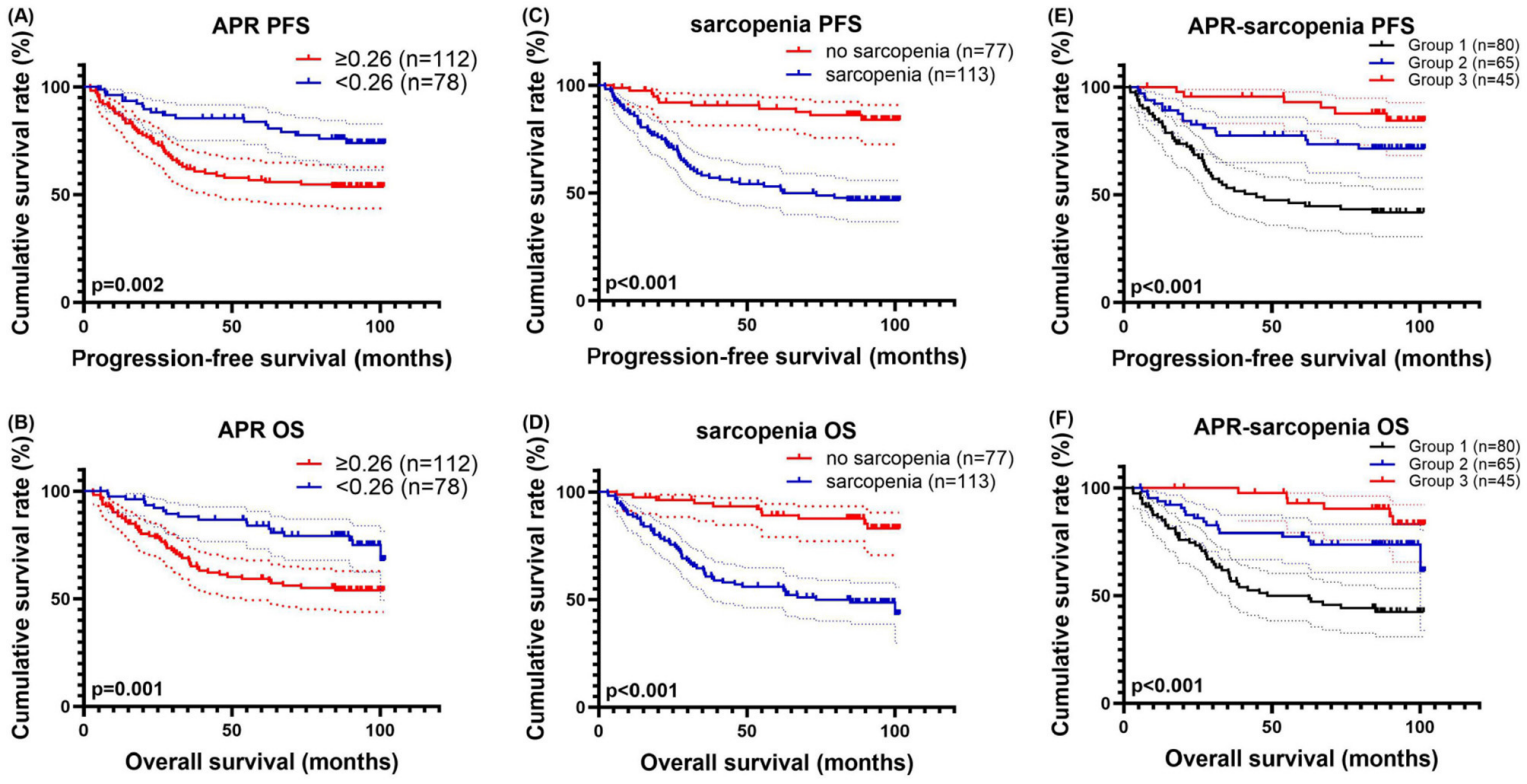
APR–related survival curve for **(A)** PFS and **(B)** OS, sarcopenia–related survival curve for **(C)** PFS and **(D)** OS, and APR–sarcopenia–related survival curve for **(E)** PFS and **(F)** OS.

We performed a survival analysis for APR due to its highest AUC compared to other hematological composite biomarkers. The optimal cut-off value for APR was determined through ROC curve analysis, yielding a value of 0.26. In this cohort, among the 78 patients with APR < 0.26, the 1-, 3-, and 5-year survival rates for PFS and OS were 96.1%, 85.3%, and 83.7% and 97.4%, 88.0%, and 83.8%, respectively. In the 112 patients with APR ≥ 0.26, the 1-, 3-, and 5-year PFS and OS rates were 86.6%, 61.9%, and 56.8% and 88.3%, 66.1%, and 59.3%, respectively. Patients with elevated APR levels experienced shorter PFS (HR = 2.261, *p* = 0.003) and OS (HR = 2.377, *p* = 0.002) (Figures 4A, B).

In this cohort, women with SMI less than 31.40 cm^2^/m^2^ and men with SMI less than 39.26 cm^2^/m^2^ were diagnosed with sarcopenia. Out of the total, 113 patients were diagnosed with sarcopenia, while 77 patients were not. The 1-, 3-, and 5-year survival rates for both PFS and OS in patients with sarcopenia were 85.7%, 58.2%, and 53.1% and 88.4%, 61.8%, and 56.1%, respectively. In addition, patients without sarcopenia had 1-, 3-, and 5-year survival rates for PFS and OS of 97.4%, 90.6%, and 89.1% and 97.4%, 94.7%, and 89.0%, respectively. Patients without sarcopenia experienced significantly better PFS (HR = 0.214, *p* < 0.001) and OS (HR = 0.214, *p* < 0.001) (Figures 4C, D).

### Survival analysis for APR-sarcopenia

Given the strong predictive ability of APR and sarcopenia, we combined them for further survival analysis. We categorized the patients into three groups: Group 1 consist ed of 80 cases, with PFS rates of 83.7%, 53.1%, and 46.1% at 1-, 3-, and 5-year, and OS rates of 86.2%, 57.9%, and 49.8%, respectively. Group 2 included 65 cases, with 1-, 3-, and 5-year PFS rates of 99.2%, 77.4%, and 77.4%, and OS rates of 93.8%, 79.2%, and 77.5%. Group 3 had 45 cases, with 1-, 3-, and 5-year PFS rates of 100.0%, 95.5%, and 92.9%, and OS rates of 100.0%, 100.0%, and 92.8%. Patients in Group 3 exhibited longer PFS (HR = 0.403, *p* < 0.001) and OS (HR = 0.394, *p* < 0.001) compared to the other groups (Figures 4E, F).

### Subgroup analysis of patients with different TNM stages

**Figure 5:**
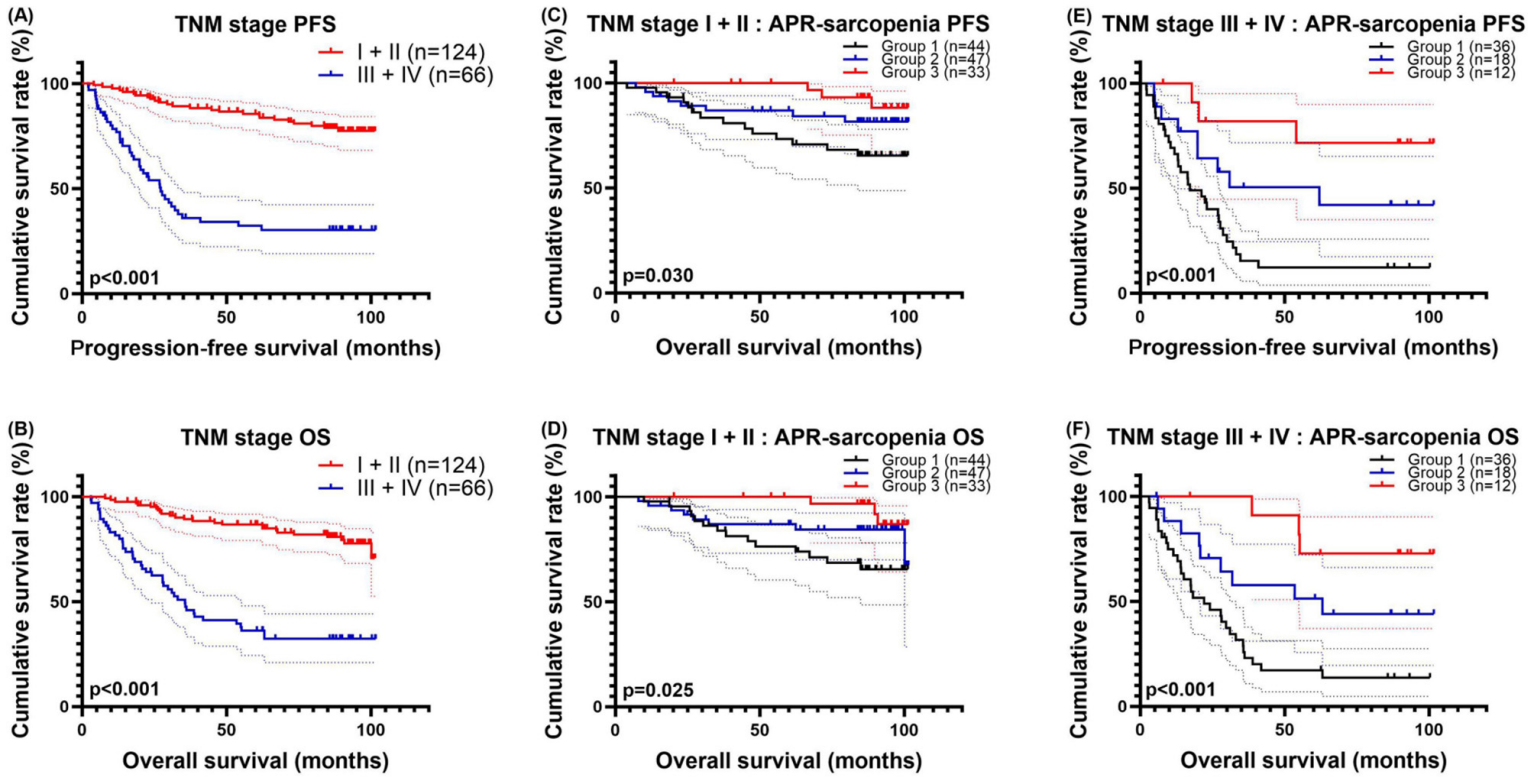
TNM–related survival curve for **(A)** PFS and **(B)** OS, APR–sarcopenia–related survival curve in early pTNM stage for **(C)** PFS and **(D)** OS, and in advanced pTNM stage for **(E)** PFS and **(F)** OS.

Additionally, we performed a subgroup analysis based on the patients' different TNM stages. We classified the 190 patients into two groups based on their TNM stages. There were 124 cases with stage I and II, with 1-, 3-, and 5-year survival rates for PFS and OS of 97.6%, 89.2%, and 85.6% and 97.6%, 89.2%, and 86.7%. Meanwhile, there were 103 patients with stage III and IV, with 1-, 3-, and 5-year survival rates for PFS and OS of 76.8%, 36.1%, and 32.3% and 81.5%, 47.7%, and 36.1%, respectively. Patients with advanced stage closely related to PFS (HR = 5.582, *p* < 0.001) and OS (HR = 5.180, *p* < 0.001) (Figures 5A, B).

In early stage group, there were 44 patients in group 1, 47 in group 2, and 33 in group 3. Group 1 consisted of 44 patients, with PFS rates of 97.7%, 83.4%, and 73.3% at 1-, 3-, and 5-year, respectively. OS rates for this group were 97.7%, 83.7%, and 76.3%. Group 2, which included 47 patients, showed PFS rates of 95.7%, 86.9%, and 86.9% at 1-, 3-, and 5-year, respectively, and OS rates of 95.7%, 86.9%, and 86.9%. Group 3, which comprised 33 patients, had PFS rates of 100.0%, 100.0%, and 100.0% at 1-, 3-, and 5-year, respectively, and OS rates of 100.0%, 100.0%, and 100.0%. Patients in Group 3 exhibited longer PFS (HR = 0.488, *p* = 0.012) and OS (HR = 0.478, *p* = 0.010) compared to the other groups (Figures 5C, D).

Among patients with TNM stages III and IV, there were 36 patients in group 1 with 1-, 3-, and 5-year survival rates of 66.3%, 15.4% and 12.3% for PFS and 71.9%, 25.9% and 17.3% for OS. At the same time, there were 18 patients in Group 2 with 1-, 3-, and 5-year survival rates of 83.0%, 50.6% and 50.6% for PFS and 88.2%, 57.8% and 51.3% for OS. In addition, there were 12 patients in Group 3 with 1-, 3-, and 5-year survival rates of 100.0%, 81.8% and 71.6% for PFS and 100.0%, 100.0% and 72.7% for OS. Patients in Group 3 also had longer PFS (HR = 0.414, *p* < 0.001) and OS (HR = 0.383, *p* < 0.001) (Figures 5E, F).

### Nomograms

**Figure 6:**
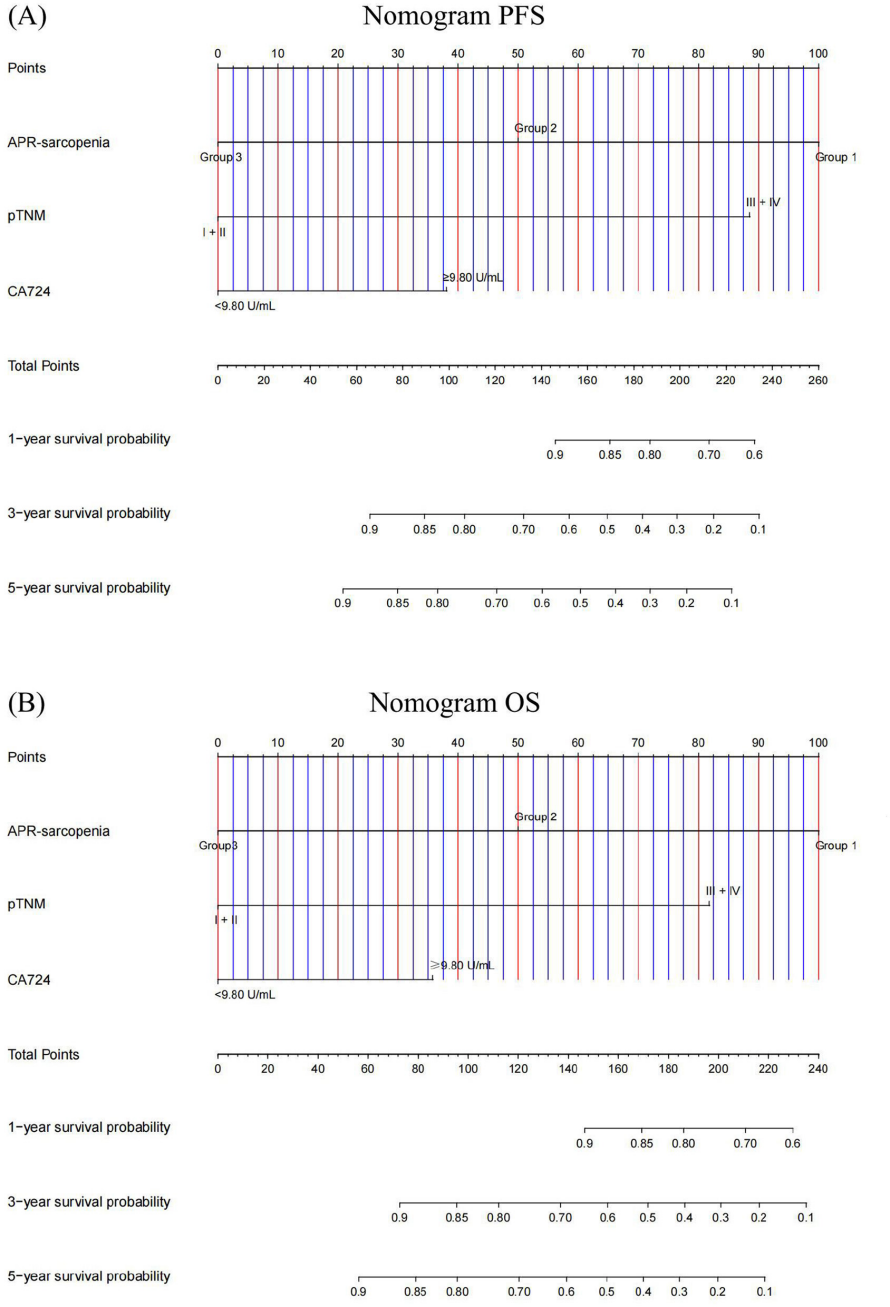
Nomogram for predicting 1–, 3–, 5–year survival probability of **(A)** PFS and **(B)** OS.

**Figure 7:**
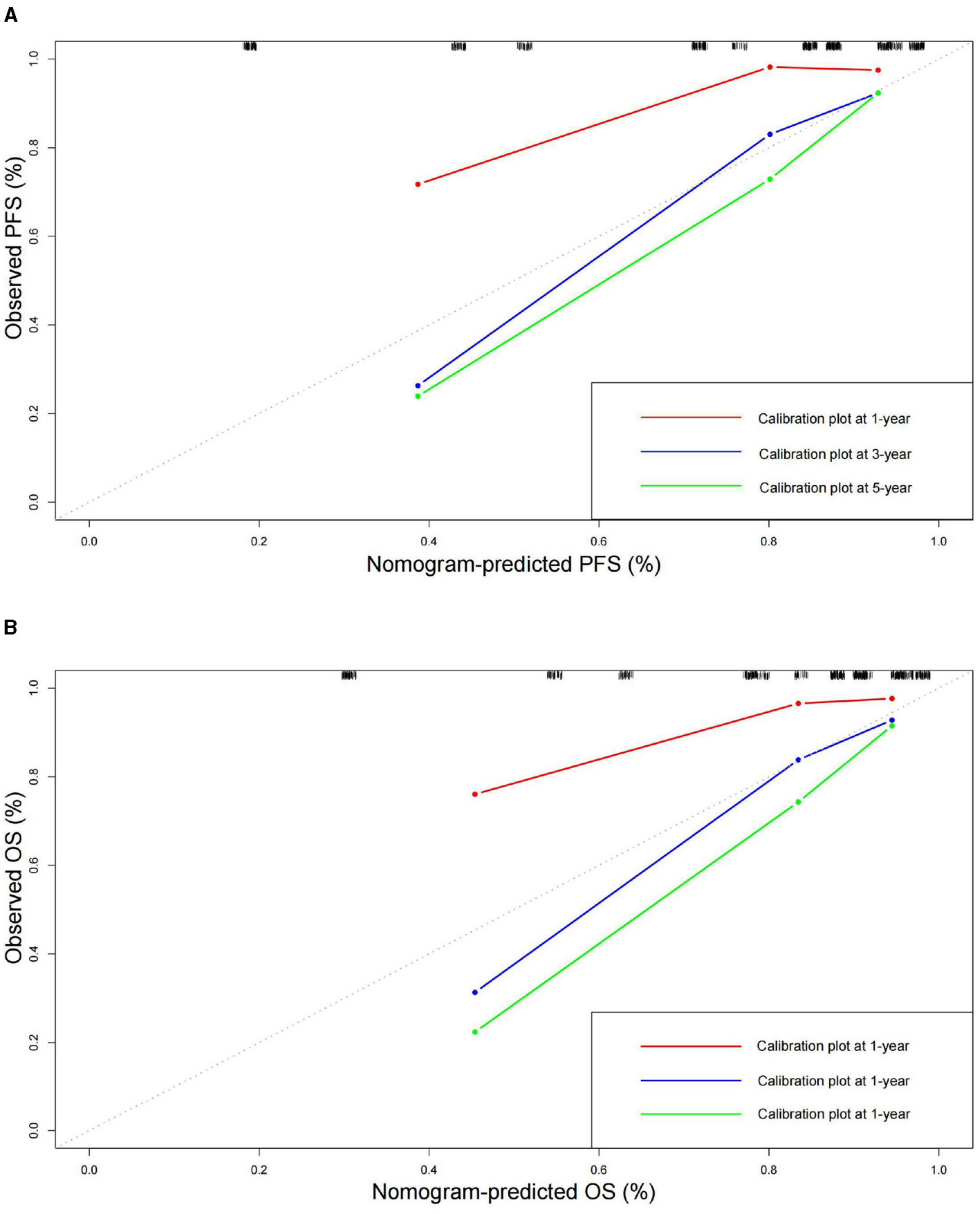
Calibration curves for predicting **(A)** PFS and **(B)** OS at 1–, 3–, and 5–year.

To further validate the prognostic significance of the combined indicator, we created nomograms to predict the probabilities of PFS and OS based on CA724, APR-sarcopenia and TNM stage. The C-index and 95% CI of the nomograms were 0.794 (95% CI: 0.743–0.845) for PFS and 0.801 (95% CI: 0.751–0.851) for OS (Figure 6). The results of calibration curves indicated good predictive accuracy of nomograms (Figure 7).

## Discussion

Given the clinical feasibility of preoperative blood testing, numerous studies have identified effective prognostic biomarkers derived from hematological parameters to predict cancer outcomes (15–17). Systemic inflammation and nutritional impairment are well-established adverse prognostic factors in cancer patients (18, 19). Alkaline phosphatase-to-prealbumin ratio was first introduced in a retrospective cohort study of 406 patients and demonstrated superior prognostic predictive performance for gastric cancer outcomes (9). An umbrella review analyzing 30 meta-analyses on sarcopenia and its association with adverse outcomes found a significant association between sarcopenia and worse prognosis across 12 types of cancer: gastric, hepatocellular, urothelial, head and neck, hematologic malignancies, pancreatic, breast, colorectal, lung, esophageal, and ovarian cancers (20). Moreover, a meta-analysis encompassing 5,421 patients undergoing abdominal surgery demonstrated that preoperative sarcopenia was significantly associated with increased all-cause mortality (21).

In our study, we assessed the predictive ability of hematological composite biomarkers by conducting ROC analysis and found that APR had the highest AUC. Sarcopenia, determined by CT imaging, not only reflects the patient's muscle mass but is also closely associated with the prognosis of gastric cancer patients and those who have undergone abdominal surgery (22). Therefore, we integrated sarcopenia with APR to enhance predictive power. The combined biomarker demonstrated superior predictive ability compared to individual application and achieved a predictive performance comparable to that of TNM staging. Cox regression analysis showed that APR-sarcopenia was an independent prognostic factor for both PFS and OS. Considering the impact of tumor staging on the prognosis of gastric cancer patients, we conducted a subgroup analysis based on different stages. APR-sarcopenia demonstrated superior predictive power in different stages, especially in the advanced stage. The nomograms incorporating APR-sarcopenia exhibited a strong alignment between the predicted and actual survival probabilities.

Alkaline phosphatase (ALP), widely distributed in the human liver, bones, intestines, kidneys, and placenta, demonstrates clinically significant elevation in conditions such as hepatic disorders, pregnancy, and bone fracture (23, 24). Notably, a retrospective cohort study of 491 gastric cancer patients identified ALP elevation as an independent prognostic biomarker, correlating with reduced overall survival, and showed significant postoperative reduction in ALP levels following curative resection (25). Studies have indicated that ALP levels are significantly elevated in certain gastric cancer cell lines and gradually increase as the disease progresses (26, 27). ALP is primarily used for diagnosing liver diseases. The liver is the most common site of distant metastasis in advanced gastric cancer, and gastric cancer patients with a more advanced clinical stage may have a higher risk of liver damage (28, 29). It is worth noting that liver injury is closely associated with sarcopenia, which may be an additional factor contributing to poor prognosis in gastric cancer patients (30). A study involving 4,732 patients showed that lower preoperative prealbumin levels in gastric cancer patients were associated with poorer prognosis (31). Prealbumin is primarily synthesized in the liver, and its concentration is influenced by liver function and nutritional status (32). Compared to albumin, prealbumin has a shorter half-life of approximately 2–3 days, making it more sensitive to recent changes in nutritional status, which allows prealbumin to serve as an early indicator of nutritional deficiency in gastric patients.

Due to the complex and varied causes of sarcopenia, few studies have explored its mechanisms. One possible reason is that gastric cancer patients often have problems with eating and may suffer from a lack of proper nutrition (33). Moreover, some patients may be unable to exercise because of the progression of the disease, weakness, and other factors. When elderly individuals had their protein intake maintained, muscle protein synthesis was still significantly reduced after staying in bed for 10 consecutive days (34). Another possible explanation is that sarcopenia acts as an indicator of intensified inflammatory response associated with cancer (35). In 2017, a study involving 2,470 patients with early-stage colorectal cancer found a significant association between sarcopenia and inflammatory status (36). Excessive production of IL-6 can impair the synthesis and dynamics of muscle mitochondria, as well as disrupt oxidative metabolism in muscle tissue (37). As part of the body's systemic inflammatory response to the tumor, proinflammatory cytokines and growth factors are released, exerting a significant catabolic impact on the metabolism, which can accelerate muscle degradation (38, 39). Moreover, recent studies suggest that differences in body composition among cancer patients may contribute to variations in how they metabolize chemotherapy drugs, leading to increased toxicity (40–42).

Notably, APR is an indicator associated with tumors, liver injury, and nutritional status. Compared to other hematological biomarkers, APR is less affected by inflammation, allowing it to demonstrate superior predictive ability in the absence of inflammatory response. APR-sarcopenia integrates the advantages of blood testing and CT imaging, reflecting the nutritional, immune, and other conditions of gastric cancer patients from multiple perspectives. As a result, it can accurately predict the prognosis of gastric cancer patients. Thanks to the application of artificial intelligence technology in imaging diagnostics, the diagnosis of sarcopenia will be further advanced, thereby promoting the clinical application of APR-sarcopenia (43).

This study has several limitation. First, it is a single-center retrospective study with limited data and relatively short follow-up, we are unable to eliminate the potential bias. Second, the cut-off values of APR and SMI were determined through ROC analysis, and currently, there is no unified standard for differentiation. In a study involving 1,167 patients with gastric cancer, sarcopenia was defined as an SMI of < 28.4 cm^2^/m^2^ in females and < 36.4 cm^2^/m^2^ in males (44). Conversely, in another study comprising 545 gastric cancer patients, sarcopenia was defined as an SMI of < 34.8 cm^2^/m^2^ in females and < 40.8 cm^2^/m^2^ in males (45). These findings indicate that the diagnostic thresholds for sarcopenia vary across different cohorts, underscoring the necessity for multicenter studies to establish standardized cut-off values to facilitate broader clinical application. The diagnosis of sarcopenia involves both the reduction of muscle mass and the decline of muscle strength. The advantage of using CT for diagnosing sarcopenia lies in its clinical convenience and its ability to directly demonstrate muscle mass. However, the limitation is that, although muscle mass is closely related to muscle strength, CT cannot directly reflect muscle strength. Finally, while this study introduces a new prognostic biomarker and highlights its prognostic value, further validation through larger prospective studies is required to confirm these findings.

## Conclusion

APR demonstrated superior predictive capability for postoperative prognosis of gastric cancer patients undergoing surgery compared to other hematological composite biomarkers. A novel composite biomarker, APR-sarcopenia, integrating preoperative CT imaging and hematological profiles, demonstrated superior prognostic predictive capability for gastric cancer patients compared to conventional biomarkers and achieved predictive performance comparable to that of TNM staging. Given its clinical feasibility, the novel composite biomarker holds significant potential for application in identifying gastric cancer patients with heightened risks of postoperative recurrence and mortality.

## Data Availability

The raw data supporting the conclusions of this article will be made available by the authors, without undue reservation.
